# Assessing psychometric properties and measurement invariance of the Sleep Quality Questionnaire among healthcare students

**DOI:** 10.1186/s40359-023-01276-2

**Published:** 2024-01-19

**Authors:** Mengyi Huang, Haiyan Ma, Karen Spruyt, Joseph M. Dzierzewski, Chen Jiang, Jiaxuan He, Nongnong Yang, Yiwei Ying, Bolanle Adeyemi Ola, Runtang Meng

**Affiliations:** 1 School of Public Health, Hangzhou Normal University, Hangzhou, 311121 Zhejiang China; 2 Engineering Research Center of Mobile Health Management System, Ministry of Education, Hangzhou, Zhejiang China; 3 Université Paris Cité, NeuroDiderot, INSERM, Paris, France; 4 The National Sleep Foundation, Washington, DC USA; 5 Department of Psychology, Virginia Commonwealth University, Richmond, VA USA; 6 Steinhardt School of Culture, Education and Human Development, New York University, New York, NY USA; 7 School of Nursing, Hangzhou Normal University, Hangzhou, Zhejiang China; 8 Department of Behavioral Medicine, Faculty of Clinical Sciences, Lagos State University College of Medicine, Ikeja, Lagos, Nigeria

**Keywords:** Sleep Quality Questionnaire, Measurement properties, Assessment instrument, Healthcare students, Observational longitudinal study

## Abstract

**Objective:**

The sleep of healthcare students is worth discovering. Mental health and self-rated health are thought to be associated with sleep quality. As such, valid instruments to assess sleep quality in healthcare students are crucial and irreplaceable. This study aimed to investigate the measurement properties of the Sleep Quality Questionnaire (SQQ) for Chinese healthcare students.

**Methods:**

Two longitudinal assessments were undertaken among healthcare students, with a total of 595, between December 2020 and January 2021. Measures include the Chinese version of the SQQ, Patient Health Questionnaire-4 (PHQ-4), Self-Rated Health Questionnaire (SRHQ), and sociodemographic questionnaire. Structural validity through confirmatory factor analysis (CFA) was conducted to examine factor structure of the SQQ. T-tests and ANOVAs were used to examine sociodemographic differences in sleep quality scores. Multi Group CFA and longitudinal CFA were respectively used to assess cross-sectional invariance and longitudinal invariance across two-time interval, i.e., cross-cultural validity. Construct validity, internal consistency, and test–retest reliability were correspondingly examined via Spearman correlation, Cronbach’s alpha and McDonald’s omega, and intraclass correlation coefficient. Multiple linear regression analysis was performed to examine incremental validity of the SQQ based on the PHQ-4 and SRHQ as indicators of the criterion variables.

**Results:**

CFA results suggested that the two-factor model of the SQQ-9 (item 2 excluded) had the best fit. The SQQ-9 scores differed significantly by age, grade, academic stage, hobby, stress coping strategy, anxiety, depression, and self-rated health subgroups. Measurement invariance was supported in terms of aforesaid subgroups and across two time intervals. In correlation and regression analyses, anxiety, depression, and self-rated health were moderately strong predictors of sleep quality. The SQQ-9 had good internal consistency and test–retest reliability.

**Conclusion:**

Good measurement properties suggest that the SQQ is a promising and practical measurement instrument for assessing sleep quality of Chinese healthcare students.

**Supplementary Information:**

The online version contains supplementary material available at 10.1186/s40359-023-01276-2.

## Introduction

Sleep health has become an important public health concern and is composed of six dimensions: regularity, satisfaction or quality, alertness or sleepiness, timing, efficiency or continuity, and duration [1]. “Sleep quality” is defined as an individual’s self-satisfaction in all aspects of sleep, and is used to refer to subtle aspects of the sleep experience, which cannot be captured by objective sleep parameters [2, 3]. To date, the concept of sleep quality does not have a consensus definition, but it is generally believed to be comprised of several factors: sleep continuity, sleep efficiency, sleep latency, sleep duration, awakening, and wakefulness after falling asleep [4, 5]. Even though the fact that polysomnographic parameters are considered the gold standard for measuring sleep [5, 6], common sentiment is that sleep quality considered as “subjective perception of sleep” [7]. In other words, a subjective evaluation of sleep quality may better explain everyday psychological and behavioral performance which is not captured by objective measurements [8].

Poor sleep quality has arisen as a widespread issue, with nearly one-third of adults claiming to experience dissatisfaction with their sleep [9, 10]. Numerous previous studies have found that university students and healthcare workers are at increased risk for sleep problems [11, 12]. Healthcare students, a subgroup of university students, have a significantly higher prevalence of poor sleep quality than non-medical students and general population [13, 14]. Healthcare students experience noticeable sleep issues which were primarily related to their intense academic requirements and high achievement expectations [15]. COVID-19 pandemic and associated lockdown may have contributed to a higher prevalence of sleep problems in healthcare students [13, 16, 17]. Despite healthcare students as a clinical or research sample have a significant risk for sleep disorders, did not lead to academic circles adequate attention [18].

Quality sleep is an essential part of a healthy lifestyle. Factors that influence sleep quality in healthcare students include physiological (e.g., age, BMI), psychological (e.g., stress, anxiety, and depression) [19–21], environmental (e.g., bedroom light, room temperature, and noise), and family/social expectations [4]. Importantly, psychological distress and sleep problems demonstrate a bidirectional relationship [17]. To mitigate negative effects of poor quality on their psychosomatic health, accurate monitoring and prompt diagnosis are vital, particularly in healthcare students [22, 23].

As noted previously, objective sleep measures have trouble applying in widespread applicability [6]. Scales for subjective measurement of sleep quality are widely used in research and clinical practice, with common scales including the Pittsburgh Sleep Quality Index (PSQI) [24], the Sleep Quality Scale [25], the Sleep Quality Index [26], and the Sleep Satisfaction Tool [27]. In China, existing sleep-related scales focused on assessing nighttime sleep or were designed for explicitly for clinical settings and patients. The assessment of sleep quality is not limited to nocturnal symptoms and includes many daytime consequences of poor nighttime sleep [28]. The Sleep Quality Questionnaire (SQQ) is a concise psychometrically sound instrument developed specifically for evaluating sleep quality across two core dimensions (*Sleep Difficulty* and *Daytime Sleepiness*) via 10 items among non-clinical populations [29].

The Chinese version of the SQQ (SQQ-C) has been translated, adapted, and validated, demonstrating equivalence between the two versions while maintaining original measurement properties [30–32]. The SQQ demonstrated a two-factor structure in both the original Japanese and Chinese versions. Credibility and applicability of the SQQ has been established via the COnsensus-based Standards for the selection of health Measurement INstruments (COSMIN) guidelines [33, 34]. Nonetheless, discriminant validitsy of the SQQ-C has not been fully established, and some discrepancies exist regarding factor loadings. The original version suggested that sleep quality measured by the SQQ was a strong predictor of general health; however, less was known about its association with anxiety and depressive symptoms. This study aims to further establish measurement properties of the SQQ among Chinese healthcare students. We examined discriminant validity of the SQQ-C with other constructs including anxiety, depression, and self-rated health. Furthermore, we performed multiple linear regression analysis to examine incremental validity of the SQQ with the Generalized Anxiety Disorder-2 (GAD-2), Patient Health Questionnaire-2 (PHQ-2), and Self-Rated Health Questionnaire (SRHQ).

## Methods

### Participants and procedures

This study focuses on freshman to junior undergraduate and freshman postgraduate students who are attending regular classes at university. College seniors are at a developmentally distinct phase of their educational journeys. Considering that seniors are facing more stressful life events such as graduation, job search, and clinical internship, as they are making a shift from student to worker, we excluded this population. More specifically, for undergraduate students: a group of students from the first to the third year of health management, nursing, pharmacy, clinical medicine, preventive medicine, and health services management majors were selected as a teaching class; and for postgraduate students: all first-year students in health management, health services management, and public health majors. All study data was collected via short paper-and-pencil questionnaires in two waves between December 2020 and January 2021 at a university in Hangzhou, China (an area at low risk for COVID-19 infection during data collection). Data collection was conducted during breaks between classes. Only full-time students attending classes on-site were recruited; students on suspension and long-term sick leave were excluded. Following the instructions of the study protocol, informed consent was obtained from each participant.

Baseline assessment (Time 1, T1) included a brief sociodemographic questionnaire and a small packet of scales, as described below. A follow-up assessment (Time 2, T2) was conducted approximately one week after T1 and included all measures at T1 except for the demographic survey. The average time interval between T1 and T2 was 7 days + 16.62 hours. The reproducibility of health measurements is optimized at intervals of 1–2 weeks [35]. Six hundred and thirty-seven (*N*_1_ = 637) and six hundred and sixteen (*N*_2_ = 616) valid data were obtained at T1 and T2, respectively. Both assessments included unique student ID of each respondent. The last author manually matched with ID across two assessment questionnaires via Microsoft Excel, thereby resulting in a matched sample (*N* = 595).

The study protocol was approved by the Institutional Review Board of Hangzhou Normal University Division of Health Sciences, China (Reference No. 20190076). The process was carried out following the principles of the Declaration of Helsinki [36].

### Measures

#### Sociodemographic questionnaire

During the baseline, participants were asked to provide demographic information, including gender, age, grade, academic stage (undergraduate, postgraduate), home location (urban, rural, suburban), being only child (yes, no), monthly household income (1 CNY ≈ 0.160 US dollars), part-time job (yes, no), physical exercise [purposeful exercise with the goal of improving health (yes, no)], hobby [regularly and frequently engage in activities or projects of their preference (yes, no)], frequency of visiting home (once per week, twice per week, once per month, once per quarter, once per semester, and once per academic year), and stress coping strategy [the most customary way of coping when faced with stress (emotion-focused, solution-focused, avoidance coping)].

To facilitate meaningful data analysis, we grouped age, monthly household income, and frequency of visiting home as follows: 1) Age: < 20 years and ≥ 20 years, 2) Monthly household income: below and above the average household income (10 000 CNY) of a three-member family in China in 2021 [37], 3) Frequency of visiting home: frequently versus occasionally.

#### Sleep Quality Questionnaire (SQQ)

The original SQQ is a 10-item scale consisting of the *Sleep Difficulty Subscale* (SDS, e.g., “I had trouble sleeping”) and *Daytime Sleepiness Subscale* (DSS, e.g., “I sometimes felt sleepy during the day”), which is used to assess subjective sleep quality over the past month in non-clinical samples [29]. Responses are recorded on a five-point Likert scale ranging from 0 to 4 (with 0 indicating “strongly disagree” and 4 indicating “strongly agree”). Global scores range from 0 to 40 with a higher score indicating poorer subjective sleep quality. Previous studies have demonstrated that the SDS was stably loaded by three items (items 1, 4, 9), while the DSS was loaded by six items (items 3, 5, 6, 7, 8, 10). Item 2 of the SQQ showed cross-loading in both the original and Chinese versions. The strong psychometric properties observed in previous studies have promoted wide usage of the SQQ-C as a practical measurement instrument in research and survey sites [30, 32].

#### Patient Health Questionnaire‑4 (PHQ‑4)

The PHQ is a self-report version of the Primary Care Evaluation of Mental Disorders (PRIME-MD) [38], both the original and derived scales are available from the ‘Patient Health Questionnaire (PHQ) Screeners website’ [39]. The PHQ-4 [40], consisting of the PHQ-2 (e.g., “Little interest or pleasure in doing things”) and GAD-2 (e.g., “Feeling nervous, anxious, or on edge”), are two extensively used ultra-short screening tools to measure depressive disorder and anxiety symptoms. In fact, the PHQ-4 was originally designed for patients, yet subsequent studies have continued to advance the validation and applicability of the PHQ-4 in the general population [41, 42]. Each item of the PHQ-4 is scored on a four-point Likert scale ranging from 0 (not at all) to 3 (nearly every day), with higher global scores being indicative of higher levels of depressive and anxiety symptoms. For the PHQ-2 and GAD-2, a scale score of ≥ 3 is recommended as a cut-off point between the normal range and possible cases of depression or anxiety, respectively.

#### Self-Rated Health Questionnaire (SRHQ)

The SRHQ is a simple questionnaire consisting of two items that assess self-rated physical health and mental health, respectively [43]. The SRHQ utilizes a five-point Likert scale from 1 (excellent) to 5 (extremely poor), with higher total scores implying poorer self-rated health status.

### Statistical Analyses

All statistical analyses were completed with R (4.1.3) and JASP (0.16.1). R packages of “*lavvan* (0.6–11)” [44], “*MBESS* (4.9.1)” [45], “*irr* (0.84.1)” [46], and “*semTools* (0.5–6)” [47] were applied. The missing rate of data was smaller than 5% (ranged from 0.168% to 1.681%), therefore the mean (continuous variables) or median (categorical variables) was used to impute missing values, treating the same methods as in our previously publications. Differences in sleep quality levels based on sociodemographic characteristics were analyzed using t-tests and ANOVA. Multiple regression models were used to examine incremental validity of the SQQ with the PHQ-4 and SRHQ as indicators of criterion variables. Following the COSMIN guidelines, different metrics were applied to assess measurement properties of the SQQ [33, 34], a brief description is provided with undermentioned items.

#### Structural validity

Based on a previous study [30], we conducted CFA on three alternative models of the SQQ: item 2 loading on the SDS (Factor 2), item 2 loading on the DSS (Factor 1), and the SQQ-9 (item 2 excluded). The weighted least squares mean and variance adjustment (WLSMV) method was used for all CFA analyses, given that the data were ordinal [48]. The indexes of goodness-of-fit used were the chi-square (*χ²*) value, *P* value, comparative fit index (CFI), Tucker-Lewis index (TLI), Akaike information criterion (AIC), Bayesian information criterion (BIC), and root means square error of approximation (RMSEA). For interpretation purposes: smaller values of AIC and BIC suggest a better model fit, CFI and TLI ≥ 0.90, RMSEA ≤ 0.08 are considered adequate and CFI and TLI ≥ 0.95, RMSEA ≤ 0.05 are considered good [49–51]. *χ²* is considered as sensitive in large samples and tends to reject the optimal model as the sample size increases, we do not use it as the only criterion for the goodness-of-fit judgment.

#### Cross-cultural validity/measurement invariance

Multi Group CFA and longitudinal CFA were conducted for testing measurement invariance (MI) of the SQQ-C for sociodemographic variables and across time intervals, respectively. In cross-population and cross-time applications of the scale, the establishment of MI means that construct measured by instrument does not change with population heterogeneity and over time. MI will be established on unbiased latent construct comparisons across groups and time. Increasingly constrained and nested models are stacked and tested against each other. That is, the SQQ has the same interpretation: the measurement structure and domain are relatively stable [52], with strict model invariance being well-validated [53].

Four nested models including configural, metric, scalar, and strict models were tested. Configural invariance requires only the same basic structural relations between observed variables. Metric invariance implies similar measurement constructs and limits factor loading to be equivalent across groups. Scalar invariance limits factor loadings and intercepts, meaning that the systematic bias of measurement content is consistent across groups and time. Strict invariance proves that group differences are caused by latent variables by limiting item residuals [54]. The fit indexes CFI, TLI, and RMSEA were applied on evaluating the goodness of model fit, changes (Δ) in CFI were used to assess whether there was invariance between progressively constrained models, where ΔCFI ≤ 0.010 was considered satisfactory [51, 55].

#### Construct/discriminant validity

Spearman correlation coefficient was calculated to evaluate construct/discriminant validity of the SQQ total and subscales with the GAD-2, PHQ-2, and SRHQ [56]. Considering the bidirectional association between sleep quality and psychosomatic health, we hypothesized that the SQQ would have a moderately strong correlation (0.30 < *r* ≤ 0.50) with the GAD-2, PHQ-2, and SRHQ [57].

#### Internal consistency and test–retest reliability

Ordinal Cronbach’s alpha and McDonald’s omega were calculated to estimate internal consistency of the SQQ and were considered good when they were equal to or greater than 0.70 [45, 55]. Test–retest reliability of the scale was assessed using intraclass correlation coefficient (ICC), with values between 0.50 and 0.75 indicative of moderate reliability, and values between 0.75 and 0.90 indicate good reliability [58]. Meanwhile, standard error of measurement (SEM) was used as a supplementary index to determine measurement accuracy in the evaluation of test–retest reliability [59].

#### Multivariate regression analyses

Sleep difficulty and daytime sleepiness were used as potential influences on negative symptoms (anxiety and depression) and self-rated health for further multiple linear regression analysis. Baseline sleep quality scores were applied to predict anxiety, depression, and self-rated health scores at follow-up to examine the incremental validity of the SQQ. Confirmation of linear relationship between variables, autocorrelation statistics were performed with the Durbin-Watson test. The variance inflation factor (VIF) was used to evaluate the multicollinearity between variables, and less than 10 was considered to be the absence of collinearity [60].

## Results

### Participants

The final 595 participants (Table 1) included in this study ranged in age from 17 to 31, with an average age of 19.857 ± 1.625 years. Among respondents, 554 (93.109%) were undergraduates and 41 (6.891%) were postgraduates. The average score of the SQQ-9, SQQ, PHQ-4, and SRHQ at each timepoint was 15.968 ± 5.449, 18.111 ± 6.102, 3.726 ± 2.390, and 4.598 ± 1.210 at T1, 16.114 ± 5.438, 18.237 ± 6.095, 3.508 ± 2.107, and 4.418 ± 1.120 at T2, separately. Table S1 (Additional file) lists the description of item and factor scores for the main variables.

**Table 1:** Sociodemographic variables and comparison of sleep quality (*N* = 595)

Variables	*N* (%)	Baseline			Follow-up		
		**Mean (SD)**	***t/F***	***P***	**Mean (SD)**	***t/F***	***P***
Gender							
Male	143 (24.034)	15.322 (5.955)	-1.630	0.104	15.448 (5.777)	-1.685	0.093
Female	452 (75.966)	16.173 (5.270)			16.325 (5.315)		
Age							
< 20 years	249 (41.849)	16.530 (5.329)	2.141	0.033*	16.924 (5.535)	3.102	0.002*
≥ 20 years	346 (58.151)	15.564 (5.507)			15.532 (5.299)		
Grade							
Freshmen	246 (41.345)	15.789 (5.659)	4.374	0.013*	15.988 (5.697)	5.988	0.003*
Sophomore	158 (26.555)	17.013 (5.310)			17.291 (5.154)		
Junior	191 (32.100)	15.335 (5.185)			15.304 (5.177)		
Academic stage							
Undergraduate	554 (93.109)	16.090 (5.350)	2.016	0.044*	16.258 (5.335)	2.381	0.018*
Postgraduate	41 (6.891)	14.317 (6.506)			14.171 (6.438)		
Home location							
Urban	227 (38.151)	15.612 (5.283)	1.816	0.164	15.938 (5.721)	0.429	0.651
Rural	221 (37.143)	15.855 (5.532)			16.063 (5.163)		
Suburban	147 (24.706)	16.687 (5.545)			16.463 (5.412)		
Being only child							
Yes	237 (39.832)	15.502 (5.641)	-1.700	0.090	15.755 (5.781)	-1.311	0.190
No	358 (60.168)	16.277 (5.304)			16.352 (5.193)		
Monthly household income							
< 10 000 CNY	245 (41.176)	16.016 (5.472)	0.181	0.857	15.947 (4.959)	-0.628	0.530
≥ 10 000 CNY	350 (58.824)	15.934 (5.441)			16.231 (5.753)		
Part time job							
Yes	101 (16.975)	17.069 (5.382)	2.236	0.026*	16.673 (5.792)	1.134	0.257
No	494 (83.025)	15.743 (5.441)			16.000 (5.362)		
Physical exercise							
Yes	296 (49.748)	15.601 (5.572)	-1.636	0.102	15.723 (5.547)	-1.750	0.081
No	299 (50.252)	16.331 (5.309)			16.502 (5.309)		
Hobby							
Yes	422 (70.924)	15.630 (5.578)	-2.370	0.018*	15.713 (5.602)	-2.826	0.005*
No	173 (29.076)	16.792 (5.042)			17.092 (4.893)		
Frequency of visiting home							
Frequently	149 (25.042)	15.671 (5.279)	-0.768	0.443	15.886 (5.539)	-0.592	0.554
Occasionally	446 (74.958)	16.067 (5.507)			16.191 (5.408)		
Stress coping strategy							
Emotion-focused	347 (58.320)	16.378 (5.184)	21.508	< 0.001*	16.398 (5.310)	8.985	< 0.001*
Solution-focused	198 (33.277)	14.359 (5.274)			15.040 (5.594)		
Avoidance coping	50 (8.403)	19.500 (5.832)			18.400 (4.798)		
GAD-2^a^							
< 3	471 (79.160)	15.030 (5.068)	-8.684	< 0.001*	15.301 (5.253)	-7.422	< 0.001*
≥ 3	124 (20.840)	19.532 (5.393)			19.202 (5.022)		
PHQ-2^b^							
< 3	486 (81.681)	15.113 (5.174)	-8.558	< 0.001*	15.313 (5.303)	-7.983	< 0.001*
≥ 3	109 (18.319)	19.780 (5.014)			19.688 (4.533)		
SRHQ^c^							
≤ 4	334 (56.134)	14.231 (5.050)	-9.427	< 0.001*	14.635 (5.441)	-7.885	< 0.001*
> 4	261 (43.866)	18.192 (5.132)			18.008 (4.820)		

*GAD* Generalized Anxiety Disorder, *PHQ* Patient Health Questionnaire, *SRHQ* Self-Rated Health Questionnaire, *SD* standard deviation, *Mean (SD)* mean scores and SDs of the SQQ-9 (item 2 excluded), *t/F* t or F value of t-test or ANOVA

^a^a score of 3 or greater implies possible anxiety symptoms

^b^a score of 3 or greater implies possible depressive symptoms

^c^a score greater than 4 implies poor self-rated health status (median score as cut point value)

^*^P < 0.05

### Structural validity

We tested three alternative models (i.e., item 2 in the SDS, item 2 in the DSS, and item 2 excluded) for CFA (Table 2). It is evident that the item 2 in the SDS model (original model) obtained an inadequate fit, while the SQQ-9 (item 2 excluded) model slightly outperformed the item 2 in the DSS model in terms of fit indices (except RMSEA). The smaller AIC and BIC values of the SQQ-9 model imply that there is greater stability and applicability of the SQQ toward Chinese healthcare students.

**Table 2:** Fit indices for three alternative CFA models of the SQQ (*N* = 595)

Model	*χ*^*2*^* (df)*	*P*	CFI	TLI	AIC	BIC	RMSEA (90% CI)
Baseline							
Item 2 in the SDS^a^	265.529 (34)	< 0.001	0.847	0.797	15,982.736	16,074.895	0.120 (0.108, 0.132)
Item 2 in the DSS^b^	104.315 (34)	< 0.001	0.953	0.938	15,821.522	15,913.682	0.059 (0.046, 0.072)
Item 2 excluded^c^	63.549 (26)	< 0.001	0.970	0.959	14,239.977	14,323.359	0.067 (0.055, 0.080)
Follow-up							
Item 2 in the SDS^a^	268.677 (34)	< 0.001	0.870	0.828	15,242.308	15,334.468	0.118 (0.106, 0.130)
Item 2 in the DSS^b^	92.785 (34)	< 0.001	0.967	0.957	15,066.416	15,158.576	0.054 (0.041, 0.067)
Item 2 excluded^c^	62.122 (26)	< 0.001	0.977	0.968	13,563.274	13,646.657	0.059 (0.047, 0.072)
Threshold	N/A	> 0.050	≥ 0.950	≥ 0.950	N/A	N/A	≤ 0.080

*CFA* confirmatory factor analysis, *SQQ* Sleep Quality Questionnaire, *SDS* Sleep Difficulty Subscale, *DSS* Daytime Sleepiness Subscale, *χ²* chi-square, *df* degrees of freedom,* CFI* comparative fit index, *TLI* Tucker-Lewis index, *AIC* Akaike information criterion, *BIC* Bayesian information criterion, *RMSEA* root mean square error of approximation, *CI* confidence interval, *N/A* not applicable

^a^item 2 belongs to the *SDS* (original structure)

^b^item 2 belongs to the *DSS*

^c^item 2 excluded from the SQQ

### Sociodemographic factors related to sleep quality

There were no significant differences in sleep quality scores between genders, but differences in the SQQ-9 scores between age, grade, and academic stage groups (Table 1). Students who had a hobby and responded to stressful events with a positive attitude had better sleep quality. Meanwhile, students with anxiety symptoms, depressive tendencies, or poor self-rated health had high scores on the SQQ-9.

### Cross-cultural validity/measurement invariance

Table 3 presents the results of examining cross-sectional measurement invariance (CMI) among different subgroups. The four models with constrained stepwise invariance assumptions were well-fitted in subgroups for both the baseline and follow-up data: CFI was from 0.953 to 0. 997, TLI was from 0.958 to 0.998, and all REMSA values were less than 0.080. Further, except that the ΔCFI of the metric model in the baseline SRHQ subgroup (-0.011), all the other ΔCFI were in threshold levels. The complete fitting information can be accessed in Additional file: Table S2. Δ*χ*² indicated no significant difference in the invariance of models in nearly all subgroups.

**Table 3:** Cross-sectional measurement invariances of the SQQ-9 (*N* = 595)

Hypothesis	Baseline							Follow-up						
	***χ***^***2***^*** (df)***	***P***	***Δχ***^***2***^*** (Δdf)***	**CFI**	**ΔCFI**	**TLI**	**RMSEA**	***χ***^***2***^*** (df)***	***P***	***Δχ***^***2***^*** (Δdf)***	**CFI**	**ΔCFI**	**TLI**	**RMSEA**
Gender (male vs. female)														
Configural	111.148 (52)	< 0.001		0.978		0.969	0.062	95.229 (52)	< 0.001		0.989		0.984	0.053
Metric	150.822 (76)	< 0.001	33.242 (24)	0.972	-0.006	0.973	0.058	109.290 (77)	0.009	20.035 (25)	0.991	0.002	0.992	0.038
Scalar	156.553 (83)	< 0.001	8.266 (7)	0.972	0.000	0.976	0.055	118.074 (84)	0.008	8.439 (7)	0.991	0.000	0.992	0.037
Strict	165.150 (92)	< 0.001	9.936 (9)	0.972	0.000	0.978	0.052	142.833 (93)	0.001	19.219 (9)	0.987	-0.004	0.990	0.043
Age (< 20 years vs. ≥ 20 years)														
Configural	112.565 (52)	< 0.001		0.978		0.969	0.063	94.722 (52)	< 0.001		0.989		0.984	0.053
Metric	132.548 (74)	< 0.001	22.035 (22)	0.978	0.000	0.979	0.052	116.055 (77)	0.003	22.204 (25)	0.990	0.001	0.990	0.041
Scalar	136.175 (81)	< 0.001	6.723 (7)	0.980	0.002	0.982	0.048	139.158 (84)	< 0.001	18.447 (7)	0.985	-0.005	0.988	0.047
Strict	157.847 (90)	< 0.001	16.489 (9)	0.975	-0.005	0.980	0.050	140.508 (93)	0.001	4.142 (9)	0.987	0.002	0.990	0.042
Grade (freshmen vs. sophomore vs. junior)														
Configural	139.330 (78)	< 0.001		0.978		0.969	0.063	124.259 (78)	0.001		0.987		0.983	0.055
Metric	189.512 (122)	< 0.001	48.25 (44)	0.976	-0.002	0.978	0.053	157.349 (120)	0.013	38.003 (42)	0.990	0.003	0.991	0.040
Scalar	206.646 (136)	< 0.001	17.12 (14)	0.974	-0.002	0.980	0.051	185.991 (134)	0.002	22.742 (14)	0.986	-0.004	0.989	0.044
Strict	233.238 (154)	< 0.001	25.082 (18)	0.971	-0.003	0.980	0.051	209.020 (152)	0.001	22.827 (18)	0.984	-0.002	0.989	0.044
Academic stage (undergraduate vs. postgraduate)														
Configural	116.681 (52)	< 0.001		0.975		0.965	0.065	87.935 (52)	0.001		0.990		0.986	0.048
Metric	131.931 (74)	< 0.001	22.768 (22)	0.977	0.002	0.978	0.051	104.062 (73)	0.010	20.579 (21)	0.991	0.001	0.991	0.038
Scalar	129.105 (81)	0.001	6.006 (7)	0.981	0.004	0.983	0.045	105.017 (80)	0.032	6.074 (7)	0.993	0.002	0.994	0.032
Strict	139.436 (90)	0.001	10.624 (9)	0.981	0.000	0.985	0.043	106.547 (89)	0.099	7.634 (9)	0.995	0.002	0.996	0.026
Part-time job (yes vs. no)														
Configural	112.565 (52)	< 0.001		0.978		0.969	0.063	87.335 (52)	0.002		0.991		0.987	0.048
Metric	132.548 (74)	< 0.001	22.035 (22)	0.978	0.000	0.979	0.052	110.009 (73)	0.003	22.219 (21)	0.990	-0.001	0.990	0.041
Scalar	136.175 (81)	< 0.001	6.723 (7)	0.980	0.002	0.982	0.048	116.311 (80)	0.005	7.369 (7)	0.990	0.000	0.991	0.039
Strict	157.847 (90)	< 0.001	16.489 (9)	0.975	-0.005	0.980	0.050	160.460 (89)	< 0.001	26.298 (9)	0.981	-0.009	0.985	0.052
Hobby (yes vs. no)														
Configural	97.438 (52)	< 0.001		0.983		0.977	0.054	76.544 (52)	0.015		0.993		0.990	0.040
Metric	124.545 (74)	< 0.001	24.819 (22)	0.982	-0.001	0.982	0.048	93.707 (73)	0.052	18.562 (21)	0.994	0.001	0.994	0.031
Scalar	128.679 (81)	0.001	6.301 (7)	0.983	0.001	0.985	0.045	96.786 (80)	0.097	4.983 (7)	0.995	0.001	0.996	0.027
Strict	146.834 (90)	< 0.001	16.169 (9)	0.979	-0.004	0.983	0.046	100.007 (89)	0.200	5.295 (9)	0.997	0.002	0.998	0.020
Stress coping strategy (emotion-focused vs. solution-focused vs. avoidance coping)														
Configural	116.077 (78)	0.003		0.983		0.976	0.050	126.775 (78)	< 0.001		0.986		0.980	0.056
Metric	142.304 (116)	0.049	32.913 (38)	0.988	0.005	0.989	0.034	189.980 (116)	< 0.001	49.605 (38)	0.978	-0.008	0.980	0.057
Scalar	157.500 (130)	0.051	14.946 (14)	0.988	0.000	0.990	0.033	205.362 (130)	< 0.001	15.871 (14)	0.978	0.000	0.982	0.054
Strict	183.809 (148)	0.024	21.953 (18)	0.984	-0.004	0.988	0.035	217.174 (148)	< 0.001	16.92 (18)	0.980	0.002	0.985	0.049
GAD-2^a^ (< 3 vs. ≥ 3)														
Configural	113.282 (52)	< 0.001		0.974		0.964	0.063	92.411 (52)	< 0.001		0.988		0.983	0.051
Metric	132.781 (75)	< 0.001	19.698 (23)	0.975	0.001	0.976	0.051	116.848 (75)	0.001	23.6 (23)	0.987	-0.001	0.988	0.043
Scalar	160.990 (82)	< 0.001	17.842 (7)	0.966	-0.009	0.970	0.057	131.597 (82)	< 0.001	11.352 (7)	0.985	-0.002	0.987	0.045
Strict	162.475 (91)	< 0.001	5.622 (9)	0.969	0.003	0.976	0.051	144.219 (91)	< 0.001	12.059 (9)	0.984	-0.001	0.987	0.044
PHQ-2^b^ (< 3 vs. ≥ 3)														
Configural	101.368 (52)	< 0.001		0.978		0.969	0.057	108.828 (52)	< 0.001		0.981		0.974	0.061
Metric	140.574 (74)	< 0.001	30.446 (22)	0.970	-0.008	0.971	0.055	145.843 (74)	< 0.001	29.661 (22)	0.976	-0.005	0.976	0.057
Scalar	152.511 (81)	< 0.001	10.341 (7)	0.968	-0.002	0.971	0.055	155.226 (81)	< 0.001	9.521 (7)	0.975	-0.001	0.978	0.056
Strict	156.096 (90)	< 0.001	7.151 (9)	0.970	0.002	0.976	0.050	166.809 (90)	< 0.001	12.053 (9)	0.974	-0.001	0.979	0.054
SRHQ^c^ (≤ 4 vs. > 4)														
Configural	122.313 (52)	< 0.001		0.970		0.958	0.068	92.377 (52)	< 0.001		0.988		0.984	0.051
Metric	169.709 (75)	< 0.001	42.357 (23)	0.959	-0.011	0.961	0.065	140.478 (74)	< 0.001	43.219 (22)	0.981	-0.007	0.981	0.055
Scalar	185.013 (82)	< 0.001	14.724 (7)	0.955	-0.004	0.961	0.065	147.331 (81)	< 0.001	8.323 (7)	0.981	0.000	0.983	0.053
Strict	199.700 (91)	< 0.001	15.206 (9)	0.953	-0.002	0.963	0.063	158.986 (90)	< 0.001	12.501 (9)	0.980	-0.001	0.984	0.051
Threshold	N/A	> 0.050	N/A	≥ 0.950	≤ 0.010	≥ 0.950	≤ 0.080	N/A	> 0.050	N/A	≥ 0.950	≤ 0.010	≥ 0.950	≤ 0.080

*SQQ-9* Sleep Quality Questionnaire (item 2 excluded), *χ*² chi-square, *df* degrees of freedom, *CFI* comparative fit index, *TLI* Tucker-Lewis index, *RMSEA* root mean square error of approximation, *Δ* change in *χ²*, *df,* and CFI, *N/A* not applicable, *GAD* Generalized Anxiety Disorder, *PHQ* Patient Health Questionnaire, *SRHQ* Self-Rated Health Questionnaire

^a^a score of 3 or greater implies possible anxiety symptoms

^b^a score of 3 or greater implies possible depressive symptoms

^c^a score greater than 4 implies poor self-rated health status (median score as cut point value)

Longitudinal measurement invariance (LMI) across time intervals (Table 4) also shows an excellent fit, with all fit indexes within critical values (CFI: 0.981–0.990, TLI: 0.982–0.989, RMSEA: 0.037–0.047, and SRMR: 0.039–0.042). The change in fit indexes were also in the acceptable range.

**Table 4:** Longitudinal measurement invariance of the SQQ-9 (*N* = 595)

Hypothesis	*χ² (df)*	*P*	Scaled chi-square difference test statistics		CFI	ΔCFI	TLI	ΔTLI	RMSEA (90% CI)	ΔRMSEA
			***Δχ***^***2***^*** (Δdf)***	***P***						
Configural	229.633 (120)	< 0.001			0.990		0.988		0.039 (0.031, 0.047)	
Metric	264.618 (145)	< 0.001	34.468 (25)	0.098	0.989	-0.001	0.989	0.001	0.037 (0.030, 0.044)	-0.002
Scalar	318.995 (152)	< 0.001	76.006 (7)	< 0.001	0.985	-0.004	0.985	-0.004	0.043 (0.036, 0.050)	0.006
Strict	369.138 (161)	< 0.001	54.163 (9)	< 0.001	0.981	-0.004	0.982	-0.003	0.047 (0.040, 0.053)	0.004
Threshold	N/A	> 0.050	N/A	> 0.050	≥ 0.950	≤ 0.010	≥ 0.950	≤ 0.010	≤ 0.080	≤ 0.015

*SQQ-9* Sleep Quality Questionnaire (item 2 excluded), *χ*² chi-square, *df* degrees of freedom, *CFI* comparative fit index, *TLI* Tucker-Lewis index, *RMSEA* root mean square error of approximation, *CI* confidence interval, *Δ* change in *χ²*, *df,* CFI, TLI, and RMSEA, *N/A* not applicable

### Construct/discriminant validity

**Fig. 1:**
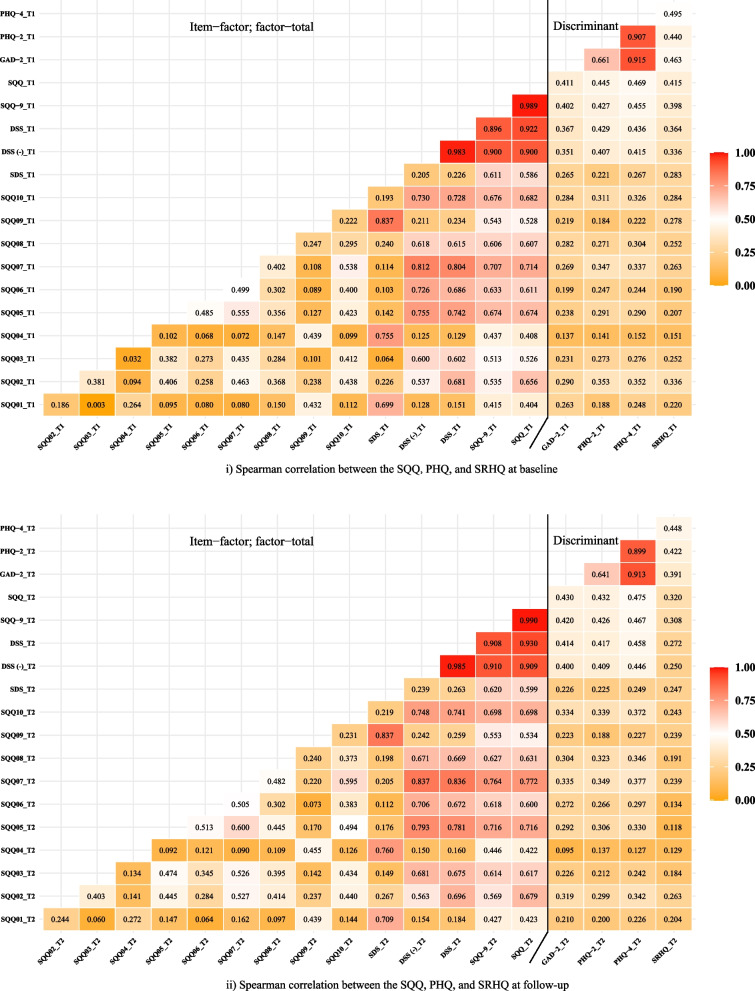
Item–factor, factor–total, and discriminant correlations between the SQQ, PHQ-4, and SRHQ (*N* = 595). *Note*: Spearman correlations, *T1* Time 1, *T2* Time 2, *SQQ* Sleep Quality Questionnaire, *SQQ01-10* item 1–10, *SDS* Sleep Difficulty Subscale, *DSS* Daytime Sleepiness Subscale, *DSS (-)* item 2 excluded from the DSS, *SQQ-9* item 2 excluded from the SQQ, *GAD* Generalized Anxiety Disorder, *PHQ* Patient Health Questionnaire, *SRHQ* Self-Rated Health Questionnaire

Parts i and ii of Fig. 1 show the results of Spearman correlation analysis between the SQQ, PHQ-4, and SRHQ items, total scores, and subdomains at T1 and T2, respectively. The coefficients on the left side of the black line are the items, factors and total correlations within the SQQ, and the coefficients on the right side of the black line are discriminative validity estimates between the SQQ with the PHQ-4 and SRHQ. Whether it is 9 items or 10 items, the correlation coefficients between the SQQ with the GAD-2, PHQ-2, PHQ-4, and SRHQ are all moderate in strength (0.30–0.50). Table S3 (Additional file) displays the results of the correlation coefficients combining the two assessments.

### Internal consistency and test–retest reliability

Although the previous CFA suggested that the SQQ-9 was a better model than the original SQQ-10, we still estimated reliability of the original scale and its subscales (Table 5). All ordinal Cronbach’s alpha and McDonald’s omega values are greater than 0.800, and ICC values suggested that the subscales and total scale have excellent reliability except for the SDS (ICC = 0.741). In this study, the GAD-2, PHQ-2, PHQ-4, and SRHQ all showed good reliability (T1: 0.814, 0.747, 0.844, 0.704; T2: 0.784, 0.743, 0.831, 0.710).

**Table 5:** Internal consistency and test–retest reliability of the SQQ (*N* = 595)

	**Ordinal Cronbach’s alpha**		**Ordinal McDonald’s omega**		**ICC (95% CI)**	**SEM**	
	**Baseline**	**Follow-up**	**Baseline**	**Follow-up**		**Baseline**	**Follow-up**
SQQ	0.823 (0.802, 0.845)	0.851 (0.833, 0.869)	0.822 (0.801, 0.843)	0.852 (0.834, 0.870)	0.816 (0.787, 0.841)	2.617	2.614
SQQ-9	0.845 (0.826, 0.865)	0.876 (0.860, 0.891)	0.849 (0.830, 0.867)	0.879 (0.864, 0.894)	0.802 (0.771, 0.829)	2.425	2.420
SDS	0.710 (0.669, 0.750)	0.733 (0.695, 0.770)	0.731 (0.695, 0.767)	0.749 (0.716, 0.783)	0.741 (0.698, 0.778)	1.236	1.181
DSS (item 2 excluded)	0.798 (0.773, 0.823)	0.831 (0.810, 0.851)	0.794 (0.769, 0.818)	0.831 (0.811, 0.851)	0.815 (0.786, 0.840)	1.895	1.890
DSS	0.860 (0.843, 0.877)	0.885 (0.871, 0.899)	0.862 (0.845, 0.879)	0.888 (0.874, 0.902)	0.829 (0.802, 0.852)	2.099	2.092

*SQQ* Sleep Quality Questionnaire, *SQQ-9* item 2 excluded from the SQQ, *SDS* Sleep Difficulty Subscale, *DSS* Daytime Sleepiness Subscale, *ICC* intraclass correlation coefficient, *CI* confidence interval, *SEM* standard error of measurement, calculated as “SD × sqrt (1-ICC)”

### Multivariate regression analyses

Multiple linear regression analysis (Table 6) was performed based on the scores of the SDS and DSS for the PHQ-4 and SRHQ. There is a linear relationship between the independent and dependent variables, and VIF value recommended little multicollinearity. Sleep quality as measured by the SQQ predicted 20.7% of anxiety and depressive symptoms and 10.8% of self-rated health, respectively. Overall, *β* weights for both daytime sleepiness and sleep difficulty scores were significant at the 0.001 level.

**Table 6:** Multiple linear regression analysis predicting negative symptoms and self-rated health (*N* = 595)

Criterion variable	Predictors	B	*SE*	*β*	*t*	*P*	95% *CI*		VIF
							**Lower**	**Upper**	
PHQ-4	R^2^ = 0.209; Adjusted R^2^ = 0.207; F = 78.325; *P* < 0.001								
	SDS	0.147	0.032	0.170	4.546	< 0.001	0.084	0.211	1.044
	DSS	0.187	0.018	0.391	10.483	< 0.001	0.152	0.222	1.044
SRHQ	R^2^ = 0.111; Adjusted R^2^ = 0.108; F = 36.929; *P* < 0.001								
	SDS	0.105	0.018	0.227	5.742	< 0.001	0.069	0.141	1.044
	DSS	0.051	0.010	0.201	5.082	< 0.001	0.031	0.071	1.044

*SDS* Sleep Difficulty Subscale, *DSS* Daytime Sleepiness Subscale, *PHQ* Patient Health Questionnaire, *SRHQ* Self-Rated Health Questionnaire, *B* unstandardized coefficients, *SE* standard error, *β* standardized coefficients, *CI* confidence interval, *VIF* variance inflation factor, *R*^*2*^ coefficient of determination

## Discussion

This study auxiliary examined measurement properties of the SQQ-C in healthcare students. Our findings indicated good structural, cross-cultural, and discriminant validity, adequate internal consistency, and stability of the SQQ-9. CMI was established based on sociodemographic variables that may affect sleep quality. LMI models across time intervals further suggested that the SQQ-9 is a promising and practical measurement instrument for assessing sleep quality. Multiple linear regression results demonstrated that sleep quality measured by the SQQ can be used to predict short-term negative symptoms (anxiety and depression) and self-rated health status.

CFA results suggested that the two-factor SQQ-9 structure fits the data best. Interestingly, such a factor structure is inconsistent with the originally proposed factor structure [29], the same factor structure appeared in our previous study using the SQQ to measure sleep quality in Chinese samples [30]. These studies yielded the SQQ-9 in suggesting that comprehension discrepancy might be due to cross-cultural differences and translation issues.

MI of the SQQ-9 held perfectly for most cross-sectional subgroups and longitudinal interval, as demonstrated by configural, metric, scalar, and strict invariances regardless of gender, age, grade, academic stage, hobby, stress coping strategy, anxiety symptoms, depressive tendencies, self-assessed health status, and across time intervals. In a previous study of healthcare students, it was found that age and gender were not significantly associated with sleep quality or daytime sleepiness [61]. This conclusion was confirmed through our invariance tests, but it was possible due to sampling homogeneity and the narrow range of age. Lastly, we showed the SQQ-9 has strict LMI across two-time intervals, indicating that the SQQ-9 is a stable scale with high reliability.

Our results, as in previous studies, revealed that sleep quality in healthcare students was moderately associated with negative mental states (anxiety and depression) [62, 63]. The findings were also consistent with a previous study on medical students in Malaysia, where daytime sleepiness was more pronounced among medical students who reported poor sleep quality and psychological distress [64].

Cronbach’s alpha, McDonald’s omega, ICC, and SEM were used to measure the reliability of the SQQ. Given that Cronbach’s alpha as an indicator of reliability is still contested [65–67], we further demonstrated high internal consistency of the SQQ and its subscales through McDonald’s omega. Both indicators demonstrated a high homogeneity between the SQQ items. Retest intervals of 1 or 2 weeks are considered typical intervals used to validate the reproducibility of health status measures used longitudinally [35, 68]. ICC is a relative measure of reliability and SEM is an indicator of absolute reliability, both showed good test–retest reliability [59]. Collectively, the evidence indicated the SQQ has good reliability.

Multiple linear regression analysis demonstrated that poor sleep quality was a predictor of negative mood (anxiety and depression) and self-rated health status. Sleep problems and mental health issues are common in modern society. Generally, good sleep can relieve psychological problems (e.g., depression, anxiety, and stress), while poor sleep may have negative effects on quality-of-life and academic performance, or vice versa [69, 70]. The relationship between sleep quality and mental health is well documented, in terms of primary prevention, thus sleep improvement represents a viable therapeutic goal that can provide significant benefits to mental health [71].

The importance of sleep to public health and the contribution of insufficient sleep to health disparities deserves to be emphasized. The development of healthy sleep education, especially early intervention on campus, contributes to the development of good sleep habits [72]. Cognitive behavioral therapy for insomnia is considered a first-line treatment for sleep improvement and standardized protocols have been developed [73]. Moreover, this has been found to be effective in reducing psychological problems and improving sleep-related quality of life [74, 75].

The SQQ-9 is a slightly short form determined from factor analysis results. However, the development of ultrashort measurement instruments is a priority and has been recognized as necessary for large-scale population screening (e.g., PHQ-2, GAD-2). In terms of sleep quality assessments, the previously developed single-item Sleep Quality Scale [76], the two-item Sleep Condition Indicator (SCI) [77, 78], and the six-item PSQI [79] suggest that short questionnaires are a potential possibility.

### Strengths and limitations

Discriminant validity and incremental validity of the SQQ-C were complemented in this study, we were able to retest almost all participants to examine the stability and establish LMI of the SQQ-C. This study has some limitations. First, generalizability may be limited by the type of university and overrepresentation of female respondents. As such, the extrapolation of conclusions may require more consideration and caution towards other samples. Second, since the survey was conducted during the COVID-19 epidemic and the university was in lockdown (students are not allowed to enter or leave the campus when it is not necessary), students may have been experiencing higher levels of psychological distress and sleep problems. Third, objective measures were not used in the study, and thus results are limited by the self-report nature of the data.

### Future directions

The stability and reliability of the SQQ-C should be examined across samples (e.g., occupational population, floating population), locations (e.g., multicenter or multilocation), and settings (e.g., communities, worksites). Similarly, the SQQ-C should be administered with other sleep assessment scales (e.g., PSQI, SCI) that yield cutoff values for good and poor sleep quality, allowing for estimation cutoff points for the SQQ-C. Additionally, including objective sleep measurements (e.g., wristwatch activity recorder, portable sleep monitoring device) in future studies could yield additional insights about sleep quality. Applying the SQQ-C routine monitoring and exploring response shifts in multi-wave would help to further investigate risk or preventive factors related to sleep health [1, 80, 81].

## Conclusions

The SQQ-C is a practical psychometrically sound instrument to assess sleep quality, including sleep difficulty and daytime sleepiness, amongst healthcare students. This study demonstrates that the SQQ-9-C has good psychometric properties and measurement invariance. Sleep quality as measured by the SQQ-9-C associated with short-term negative symptoms (i.e., anxiety and depression) and self-rated health status. Our previous and current findings combined suggests that the SQQ-C can be used to accurately measure sleep quality in community and research settings.

### Supplementary Information

**Additional file 1: Table S1. **Score distribution for each item and factor of the SQQ, PHQ-4, and SRHQ (*N* = 595). **Table S2.**  Cross-sectional measurement invariances of the SQQ (*N* = 595). **Table S3.** Spearman correlation of the SQQ with the PHQ and SRHQ across two assessments (*N* = 595).

## Data Availability

All data generated or analyzed during this study are not publicly available due to restrictions imposed by the ethics committee. The dataset supporting the conclusions is available upon reasonable request to the last author. Anyone interested in using the formatted SQQ-C and its scoring rubric should be directed to the last author when ready to initiate research collaboration.

## Abbreviations

AIC: Akaike information criterion
BIC: Bayesian information criterion
CFA: confirmatory factor analysis
CFI: comparative fit index
CMI: cross-sectional measurement invariance
COSMIN: COnsensus-based Standards for the selection of health Measurement INstruments
COVID-19: Corona Virus Disease 2019
DSS: Daytime Sleepiness Subscale
GAD: Generalized Anxiety Disorder
ICC: intraclass correlation coefficient
KMO: Kaiser–Meyer–Olkin
LMI: longitudinal measurement invariance
MI: measurement invariance
PHQ: Patient Health Questionnaire
PRIME-MD: Primary Care Evaluation of Mental Disorders
PSQI: Pittsburgh Sleep Quality Index
RMSEA: root means square error of approximation
SCI: Sleep Condition Indicator
SD: standard deviation
SDS: Sleep Difficulty Subscale
SEM: standard error of measurement
SQQ: Sleep Quality Questionnaire
SRHQ: Self-Rated Health Questionnaire
T1: Time1, Baseline
T2: Time2, Follow-up
TLI: Tucker-Lewis Index
VIF: variance inflation factor
WLSMV: weighted least squares mean and variance adjustment
